# Detection of differences between Amyloid‐Positive and Negative Participants based on Their Driving Behavior during On‐Ramp Merging

**DOI:** 10.1002/alz.089345

**Published:** 2025-01-03

**Authors:** Sai Santosh Reddy Danda, Yi Lu Murphy, Amanda Cook Maher, Carol C Persad, Jordan R Bross, Emma Flynn, Robert A. Koeppe, Bruno Giordani

**Affiliations:** ^1^ University of Michigan‐Dearborn, Dearborn, MI USA; ^2^ University of Michigan ‐ Dearborn, Dearborn, MI USA; ^3^ University of Michigan, Ann Arbor, MI USA; ^4^ University of Michigan Medical School, Ann Arbor, MI USA

## Abstract

**Background:**

Cognitive changes affecting performance are subtle in early stages of Alzheimer’s Disease (AD) and may emerge only with more complex tasks. Driving is a highly challenging instrumental activity of daily living, requiring higher order integration of cognitive skills. For example, driving on freeway entrance ramps requires heightened cognitive engagement such as rapid responses to fast‐emerging traffic and sudden speed changes, combining sensory processing and manipulative actions. This study analyzes quantitative attributes related to driving behaviors and physiological responses during freeway on‐ramp, merging, and post‐merge stages from fixed‐course drives taken by older amyloid‐positive and amyloid‐negative participants.

**Method:**

We analyzed data from 21 amyloid‐positive (66‐81 years old) and 21 amyloid‐negative (65‐85 years old) consensus diagnosed cognitively normal participants in the University of Michigan’s Driving and Physiological Responses study. All 42 drivers navigated the same freeway on‐ramp, recording vehicle signals, physiological signals, and videos. Focus was on the on‐ramp, divided into ROI1 (On‐Ramp), ROI2 (Acceleration Lane), and ROI3 (10 seconds after merge). Nine driving attributes, including average speed, acceleration, and physiological signals (Heart Rate, Electrodermal activity, Blood Volume Pulse, and Skin Temperature), were assessed in each ROI. Group differences between amyloid‐positive and negative individuals were analyzed via independent sample *t*‐tests or Mann Whitney U tests, as appropriate.

**Result:**

In ROI1 amyloid‐positive participants exhibited higher average speeds (*T* = 2.15, *p* = 0.03) and lower speed changes (*U* = 103, *p =* 0.003) compared to amyloid‐negative participants. ROI2 analyses revealed increased speed variability (*T* = 2.79, *p* = 0.007) and average acceleration (*U* = 138, *p* = 0.04) in amyloid‐positive participants. Amyloid positive participants also trended toward traveling further distance in the ROI2 acceleration lane (*T* = 1.73, *p* = 0.09) and toward having increased average heart rate in ROI3 (*U* = 152, *p* = 0.08).

**Conclusion:**

These results offer insights into possible cognitive‐based decision‐making differences and potential physiological markers for early cognitive decline during challenging real‐world driving activities such as on‐ramp merging in persons at high risk for cognitive decline and AD. These findings deepen our understanding of the nuanced relationship between cognitive factors and driving behaviors. Future experiments aim to classify groups based on naturalistic drive on‐ramp merging using machine learning for more accurate classification.

